# The Effect of the Osmotically Active Compound Concentration Difference on the Passive Water and Proton Fluxes across a Lipid Bilayer

**DOI:** 10.3390/ijms222011099

**Published:** 2021-10-14

**Authors:** Magdalena Przybyło, Dominik Drabik, Joanna Doskocz, Aleš Iglič, Marek Langner

**Affiliations:** 1 Department of Biomedical Engineering, Wroclaw University of Science and Technology, Grunwaldzki 13, 50-377 Wrocław, Poland; magdalena.przybylo@pwr.edu.pl (M.P.); dominik.drabik@pwr.edu.pl (D.D.); marek.langner@pwr.edu.pl (M.L.); 2 Lipid Systems sp. z o.o., Krzemieniecka 48C, 54-613 Wrocław, Poland; 3 Laboratory of Cytobiochemistry, Faculty of Biotechnology, University of Wrocław, F. Joliot-Curie 14a, 50-383 Wrocław, Poland; 4 Laboratory of Physics, Faculty of Electrical Engineering, University of Ljubljana, Tržaška 25, SI-1000 Ljubljana, Slovenia; ales.iglic@fe.uni-lj.si; 5 Laboratory of Clinical Biophysics, Department of Orthopaedics, Faculty of Medicine, University of Ljubljana, Zaloška 9, SI-1000 Ljubljana, Slovenia

**Keywords:** lipid bilayer, water transport, proton transport, water activity, membrane mechanics

## Abstract

The molecular details of the passive water flux across the hydrophobic membrane interior are still a matter of debate. One of the postulated mechanisms is the spontaneous, water-filled pore opening, which facilitates the hydrophilic connection between aqueous phases separated by the membrane. In the paper, we provide experimental evidence showing that the spontaneous lipid pore formation correlates with the membrane mechanics; hence, it depends on the composition of the lipid bilayer and the concentration of the osmotically active compound. Using liposomes as an experimental membrane model, osmotically induced water efflux was measured with the stopped-flow technique. Shapes of kinetic curves obtained at low osmotic pressure differences are interpreted in terms of two events: the lipid pore opening and water flow across the aqueous channel. The biological significance of the dependence of the lipid pore formation on the concentration difference of an osmotically active compound was illustrated by the demonstration that osmotically driven water flow can be accompanied by the dissipation of the pH gradient. The application of the Helfrich model to describe the probability of lipid pore opening was validated by demonstrating that the probability of pore opening correlates with the membrane bending rigidity. The correlation was determined by experimentally derived bending rigidity coefficients and probabilities of lipid pores opening.

## 1. Introduction

The osmotic pressure and pH are critical homeostatic parameters meticulously maintained on the cellular level [1]. Whereas osmotic pressure is believed to be constant within the cell volume, the pH may vary between membranous organelles as required by specific local metabolic activities [2].

The main contributors to the osmotic pressure are water soluble low molecular weight compounds. Their concentrations are instantaneously adjusted by membrane transporters, whereas overall osmotic balance is facilitated by passive and/or facilitated passive water transports [3]. The flux of protons between various cellular compartments, on the other hand, is usually facilitated by active transport by dedicated pumps and/or transporters [4]. The passive proton flux across a biological membrane is also possible, but it is orders of magnitude slower than that of water [5]. It has been postulated that in biological systems protons are redistributed by the Grotthuss mechanism, which requires the coordinated movement of water molecules [6]. Therefore, the passive dissipation of the membrane proton gradient requires the existence of water bridges connecting aqueous phases. The lipid bilayer is the backbone of all biological membranes, ensuring their structural continuity and providing the necessary and universal scaffold for many metabolically important processes. The biological membrane lateral integrity is the main assumption of most experimental models of artificial and biological membranes [7,8,9]. The assumption of a lipid bilayer integrity is challenged by its high permeability to water [10,11,12]. To resolve the discrepancy, a different conceptual model is required. Numerous studies have shown that the lipid bilayer is not structured as a hydrophobic slab but is a highly structured and dynamic supramolecular aggregate [13]. The dynamic nature of the lipid bilayer is necessary for the variety of biologically relevant processes, including lateral sorting of lipids and/or membrane associated proteins [14,15,16], topological transformations caused by budding [17] and fusion processes [18,19,20] as well as adsorption and transmembrane equilibration of polar or amphiphilic molecules [21,22,23,24,25]. The simplistic membrane model assumes its homogeneity and continuity are not capable to deliver quantitative measures of such complex processes. The soft matter physics approach, where the lipid membrane is perceived as a bilayer structure and can be described in terms of Helfrich’s model [26], is much better suited for that purpose. In the approach, the bilayer structure deformation is characterized by two principal local curvatures *C*_1_ and *C*_2_ and three phenomenological parameters: the bending rigidity ($\kappa_{b}$), the Gaussian elastic modulus ($\kappa_{G}$) and the spontaneous curvature (*C*_0_) [8,26]:

(1) $$E=\frac{1}{2}\kappa_{b}{\left(C_{1}+C_{2}-C_{0}\right)}^{2}+\kappa_{G}C_{1}C_{2}$$

In such representation, $\kappa_{G}$ can be associated with the propensity for the transmembrane pore formation [27,28,29]. Pores as a topological membrane feature have been postulated previously as defects that accompany lipid rafts and other lateral heterogeneities and non-homogeneous distributions of membrane components [29,30,31,32,33,34]. Later, using computer simulations, the existence of the transient pores even in uniform lipid bilayers have been postulated [7,35]. The lipid pores are topological structures, which conveniently describe high water and proton permeabilities, the unassisted flip-flop of lipids or membrane crossing by highly hydrated amphiphiles [9,22,36,37]. At first, lipid pores were correlated exclusively with external factors, such as an electric field [38], membrane inclusion [30,31,39], electrostatics of the membrane in the contact with inner and outer electrolyte solution [30] or mechanical stress [11,22,40]. The spontaneous lipid pore formation in unstressed membranes have not been considered for some time as a viable possibility. However, it has been shown that lipid pores are present in membranes containing oxidized lipids, therefore, indicating that the lipid pore formation can be considered as an intrinsic property of a lipid bilayer [41], related to intrinsic shapes of lipid lipids and other molecules in the membrane [30,31]. In order to confirm the dependence of the occurrence of lipid pores on the membrane, lipid composition should be demonstrated. In the paper, we present experimental results, which show the dependence of water and proton permeability on membrane lipid composition. In addition, it has been shown that there is a correlation between the time needed for pore opening on the membrane bending rigidity of the membrane. The correlation indicates that the spontaneous lipid pore opening can be parametrized using the soft-matter approach as described by the Helfrich model. Using the experimental data, molecular processes leading to a lipid pore formation, which accompany water flow across the lipid bilayer, are proposed. Specifically, the lipid pore opening was associated with the water activity difference, whereas the volumetric flow of water with osmotic pressure difference.

## 2. Result and Discussion

**Figure 1:**
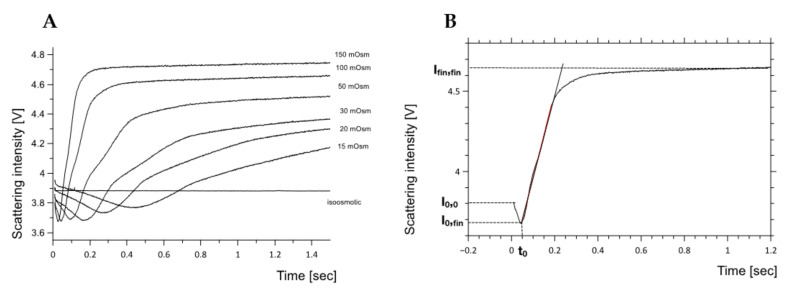
(**A**) Light scattering kinetics of egg-PC liposomes exposed to various osmotic pressure differences, collected shortly after a vesicle suspension had been mixed with KCl solution. The straight horizontal line was obtained at isosmotic condition indicating the intensity level of the scattered light when vesicles are invariant with time. (**B**) The illustration of the vesicle shrinkage kinetics and their division into three stages: the initial decrease of the light scattering intensity (stage I) doe to the outer aqueous phase equilibration, next due to the water outflow (stage II) the inner-vesicle aqueous phase equilibrates (stage III). The parameterization of liposome shrinkage kinetics was achieved by selecting the average slope determined from stage II and t_0_ as the time needed to reach minimum of the scattered light intensity (the duration of stage I).

The effect of lipid bilayer properties on water flux has been frequently measured using the method based on the self-quenching of carboxy-fluorescein dye encapsulated inside lipid vesicles [42]. However, this method requires certain assumptions, which are difficult to satisfy or verify in a real experimental setup [43]. This includes the assumption of inability of the fluorescent dye to cross the lipid bilayer barrier (not in accordance with some experimental observations [44]) and its negligible effect on the value of the osmotic pressure and/or pH inside vesicles. The later condition can be seriously compromised due to the requirement that the intra-vesicle fluorescent dye concentration is sufficiently high so the self-quenching effect can be achieved. This problem can be somewhat reduced by limiting the viable experimental data to few initial seconds following the exposure of vesicle to the osmotic pressure gradient and/or application of relatively high values of the osmotic pressure differences, therefore, making the effect of the fluorescent dye concentration negligible (>200 mOsm) (see for example Mathai et all [45]). The other serious difficulty lays in the poor reproducibility of the liposomes loading process, which is required to ensure the identical dependence of the fluorescence intensity on fluorescent dye concentration inside vesicles. The other commonly applied approach is to monitor the lipid vesicle shrinkage using the intensity of scattered light [41]. The experimental system employed in the presented studies consists of lipid vesicles formed in a solution characterized by low osmotic pressure. Then vesicles have been exposed to the high osmotic pressure solution and the resulting shrinkage kinetic was monitored. Since the osmotic pressure of the inner-vesicle aqueous phase is negligible, therefore, the osmotic pressure difference across the membrane remains constant throughout the duration of the experiment. The effect of buffer and change of the lipid bilayer surface were also neglected in the analysis. Therefore, the water flux follows the subsequent relation [46]:

(2) $$\frac{dV}{dt}=-AV_{w}P\Delta \pi$$

where A stands for the vesicle surface area, P for the water membrane permeability, V_w_ for the molar volume of water (V_w_ = 0.018L/mol), and Δπ for the osmotic pressure difference across the membrane. In such case, according to Equation (1), the water flux should remain constant in time, meaning that the dependence of the vesicle volume on time should follow the straight line with the slope proportional to the product of the salt concentration outside the vesicle and the membrane permeability coefficient [47]. Examples of experimental kinetic curves collected for egg-PC liposomes exposed to different osmotic pressure differences are presented in Figure 1A. All curves have a complex character, contrary to the prediction of the simplistic model represented by Equation (1). The intensity of the light scattered by vesicle suspension depends on the number of liposomes present in the optical path, liposome size distribution and optical properties of three distinct regions: outer aqueous phase, lipid bilayer and the inner aqueous phase. The light is scattered most effectively at the interfaces separating aqueous phases from hydrophobic core of the lipid bilayer [48]. During the stopped-flow experiment, when lipid suspension is mixed with the solution-containing compound of interest, the outer aqueous phase is instantaneously changed followed by alteration of the adjacent surface of the lipid bilayer. The mixing of the vesicle suspension with the concentrated solution will last for a few milliseconds and overlaps with dead time of the instrument. Therefore, the initial decrease of the scattered light intensity reflects changes in the restructuring (reorganization) of the outer interface of the lipid bilayer (Figure 1B). The minimal value of the scattered light intensity (I_0,fin_) following mixing corresponds to the situation where two interfaces of the lipid bilayer are exposed to different aqueous solutions and the vesicle volume is not yet altered. Next, the liposome suspension will progress towards the equilibrium state, in which the osmotic pressures difference will be balanced by the membrane resilience to deformation. The progress of the vesicle suspension, containing exclusively osmotically active compounds, towards the equilibration is possible only thanks to the osmotically driven water flux. The final level of the scattered light intensity (I_fin,fin_) will reflect the final equilibrium reached by the liposome suspension.

**Figure 2:**
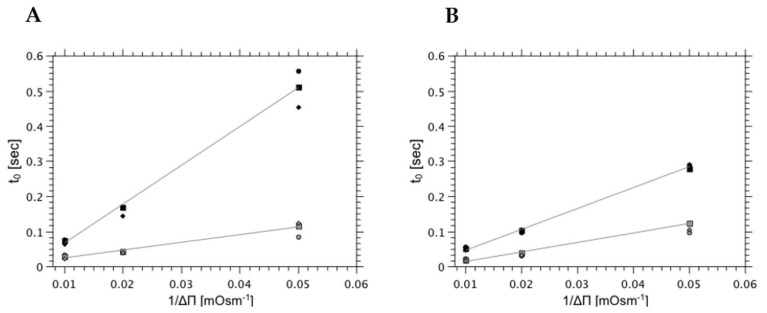
The change of the delay time as a function of 1/Δπ measured with different osmotically active compound (KCl (-●-), NaCl (-■-) or sucrose (-◆-)) at two different temperatures (15 °C—black symbols, 40 °C—open symbols) determined for (**A**) egg-PC and (**B**) egg-PC/lysoPC (85:15) liposomes.

To quantitate the effect of the osmotic pressure difference on the water flux, the perception of the experimental model has been modified. Figure 1B schematically shows the typical dependence of the light scattering on time following vesicles exposure to the osmotic pressure difference. The literature data [48] shows that the scattered light intensity of the vesicle suspension depends predominantly on the optical properties of membrane interfaces influenced by the local surface charge distribution and spatial dependent orientational ordering of water dipoles [44,47]. In other words, a change in membrane refractive index of about 0,7 results in a larger scattering intensity change than doubling the average vesicle diameter from 80 to 200 nm. The quantitative evaluation of experimental curve is based on the assumption that the water flows through the spontaneously formed lipid pores without affecting the global lipid bilayer properties excluding topology. This assumption extends the lipid bilayer description from a static barrier (described by Equation (1)) into a more accurate representation where the membrane is considered as a dynamic fluid material. The experimental curves can be sectioned into three stages; during the initial stage (I), the intensity of scattered light drops, reflecting the reorganization of the lipid bilayer outer interface and tome required for the formation of lipid pore(s). The acquired data shows that the process is much slower than the dead time of the instrument and depends on experimental conditions. It was assumed that during that time the lipid bilayer changes to facilitate the water flow. The duration of this phase equals “t_0_” and should depend on the intrinsic bilayer properties including the propensity for the lipid pore formation (Figure 2). The probability of a pore opening depends both on an intrinsic (lipid composition, intrinsic shapes of lipids, electric charge and electric dipole moments of lipids, dynamics of lipid molecules and the bending rigidity of the membrane) [30,46,49] and extrinsic (electric field or mechanical stress [50,51,52]) factors. Lipid pore(s) formation will facilitate the passive water flux described by the equation [27]. The water flow out of vesicles alters their properties (including organization of inner interface and aqueous phase) as demonstrated by the rising intensity of the scattered light. The final intensity of the light scattered by the vesicle suspension depends predominantly on combined properties of inner and outer interfaces at the final equilibrium, where the balance between the osmotic pressures, membrane mechanical resilience and thermal fluctuations, is reached. Such interpretation of experimental data leads to the conclusion that the light scattering change (I_fin,fin_−I_fin,0_) can be correlated with the membrane resilience to deformation. The bending rigidity coefficients for all studied membranes were measured using the fluorescence flicker noise spectroscopy. To account for the membrane reorganization following its exposure to the osmotic imbalance, Equation (1) was modified as follows:

(3) $$\frac{dV}{AV_{w}dt}=\left\{\begin{array}{rr} 0, & t<t_{0}\\ P\Delta \pi , & t\geq t_{0} \end{array}\right.$$

Equation (2) accounts for the two-stage kinetics of the osmotically driven scattered light intensity change. The kinetic curve can be parameterized using two arbitrary selected quantities: the time when the deflection point occurs (t_0_), and the rate of liposome volume change reflecting water outflow. The suspension of vesicles exposed to the osmotic pressure difference can be also analyzed using equilibrium quantities; the relative scattered light intensity associated with the alterations of lipid bilayer interfaces (I_0,fin_ and I_fin,fin_) as presented in Figure 1B. The deflection point can be correlated with the outer interphase reorganization, caused by the appearance of the osmotically active compound (salt or sucrose) followed by the membrane topological alteration leading to the lipid pore formation. Consequently, the delay time of the deflection point (t_0_) depends on the membrane resilience to reorganization and is proportional to 1/Δπ (Figure 2). The position of the deflection point (t_0_) is influenced mainly by the lipid bilayer dynamics, which is affected by thermal conditions of the sample and by the composition of the lipid bilayer. Figure 2 shows the dependence of time required to reach deflection state on the temperature for two different membrane compositions (left panel (A)—egg-PC liposomes and right panel (B)—egg-PC/lysoPC, 85:15, liposomes) where the water outflow was induced by KCl (-●-), NaCl (-■-) or sucrose (-◆-) concentration gradients.

**Figure 3:**
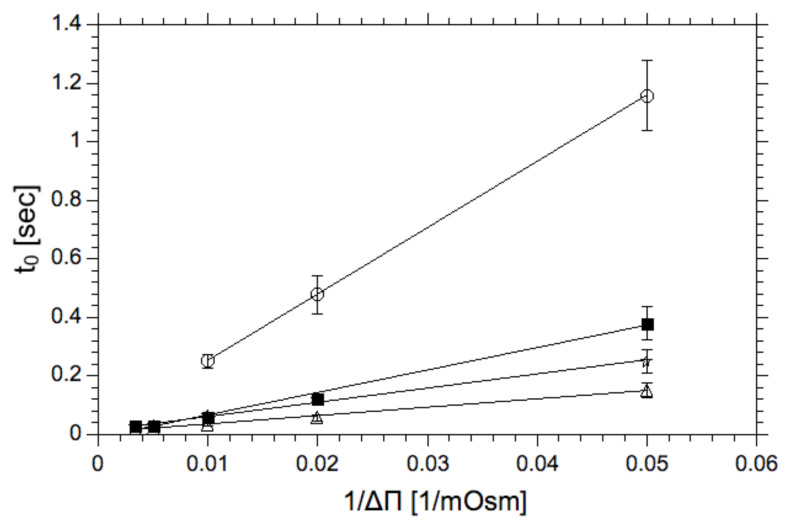
The dependence of the parameter t_0_ (as defined on Figure 1) on the osmotic pressure difference for membranes formed from various mixtures of lipids; (◯)—egg-PC/cholesterol 70/30 mol%, (△)—egg-PC/lysoPC 70/30 mol% and (✯)—egg-PC/PE 70/30 mol%. T = 25 °C.

Presented data shows that the membrane reorganization time, following the osmotic compound addition, decreases at the elevated temperature. This result can be easily explained by the increased mobility of lipids in the membrane (material softening). The other observation is that at 15 °C the lipid bilayer containing lyso-lipids (15 mol%) equilibrates much faster than membranes formed from egg-PC alone. The difference is less prominent at higher temperature (40 °C) where, in both cases, molecular mobilities are equally high with a high propensity to form surfaces with positive spontaneous curvatures, therefore, enhancing the plasticity of the lipid bilayer [53]. The dependence of the position of the deflection point on the osmotic pressure difference is presented here for the first time and demonstrates that osmotic imbalances between membrane interfaces induced its structural instability, which may lead to pore formation and enhanced water permeability. Interestingly, the position of the deflection point does not depend on the compound used for the generation of the osmotic pressure difference, showing that the membrane reorganization is not caused by the solute but rather on reduced water activity in concentrated solutions [54]. The osmotic imbalance, as shown in Figure 3, affects the lipid bilayer dynamics (the dependence of t_0_ on 1/Δπ) regardless on its lipid composition, albeit to a different degree. In all cases rising osmotic pressure difference reduces the value of parameter t_0_. As expected, the membrane containing 30 mol% of cholesterol reduces the molecular mobility of lipid molecules (membrane softness) as demonstrated by elevated t_0_ values. This result is in good agreement with data presented by others that the cholesterol stabilizes the structure of the lipid bilayer hence reducing the lipid bilayer permeability [55,56]. This result is consistent with the observations presented in the literature about lyso-lipids. Compounds capable of destabilizing the structure of the lipid bilayer should have the opposite effect. Both lyso-lipid and phosphatidylethanolamine (PE) change lipid organization, enhancing the propensity for the lipid pores formation [33,41,57].

**Figure 4:**
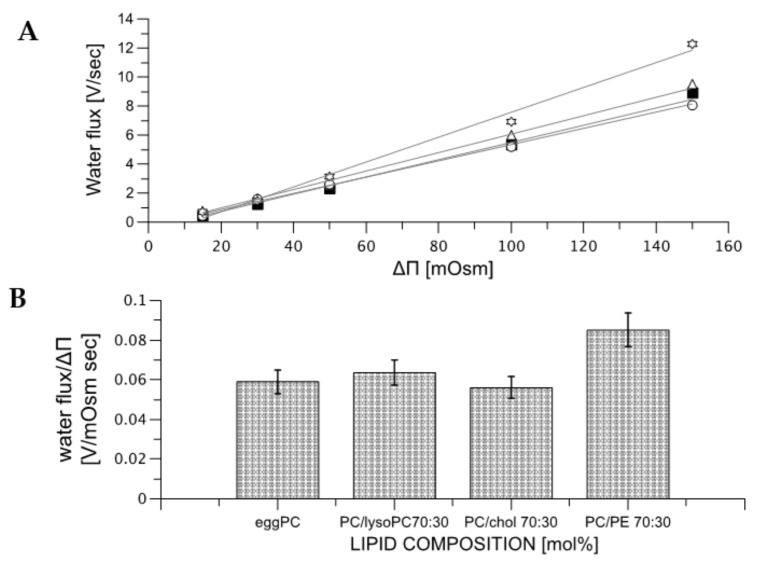
Panel (**A**) presents the water flux as a function of the osmotic pressure difference for membranes formed from different mixtures of lipids; (◯)—egg-PC/cholesterol 70/30 mol%, (△)—egg-PC/lysoPC 85/15 mol%, (▽)—egg-PC/lysoPC 70/30 mol% and (✯;)—egg-PC/PE 70/30 mol%. Panel (**B**) visualize the quantitative change of water flux (slopes for curves from panel (**A**) for selected membrane compositions).

When the lipid bilayer reorganization is completed, following addition of the osmotically active compound, the water starts to flow indicating the lipid pores opening. Figure 4 shows the dependence of the size of water flux on the osmotic pressure difference, determined for vesicles formed from phosphatidylcholine alone and these modified with cholesterol, lysoPC and PE. Data presented in Figure 4 shows that the size of the water flux increases with rising osmotic pressure difference but is practically invariant on the lipid composition of the membrane. Only the presence of phosphatidylethanolamine results with somewhat higher water fluxes.

**Figure 5:**
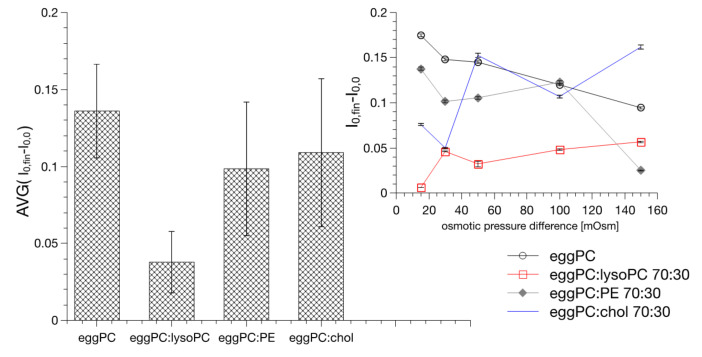
The effect of lipid composition of liposomes on the averaged values of initial drops of light scattering intensities.

**Figure 6:**
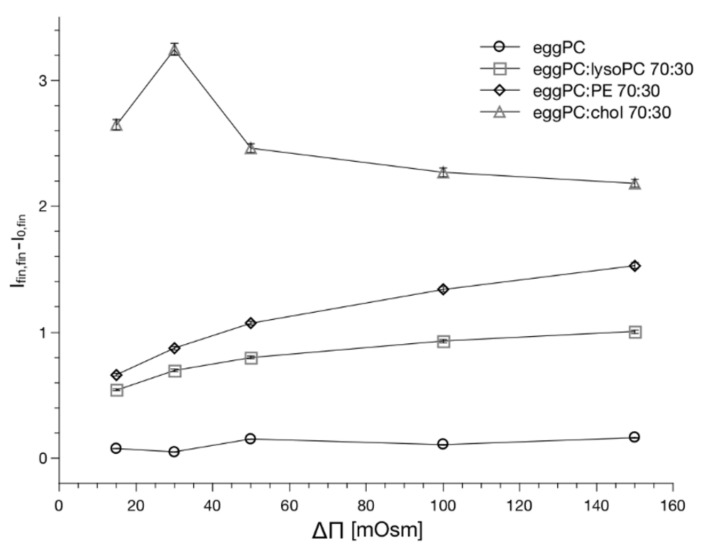
The effect of the osmotic pressure difference and lipid bilayer composition on the changes of the scattered light intensities induced by the water outflow from vesicles.

Equation (2) reduces to Equation (1) when the osmotic pressure difference is sufficiently high, i.e., above 200 mOsm so the t_0_ <10 msec (the value smaller than the dead-time of the instrument). Studies of water flow through the model lipid bilayers are typically performed at high osmotic pressure differences (typically higher than 200 mOsm). At high osmotic pressure differences, it has been sufficient to treat the lipid bilayer as a uniform and structureless hydrophobic slab when determining the osmotically driven water flow [45,58]. When approaching the equilibrium, the reduced vesicle inner volume will change the activity of remaining entrapped water as well as membrane topology. These changes will affect the optical properties of the liposome suspension [48]. Consequently, the parameters (I_0,fin_−I_0,0_) and (I_fin,fin_−I_0,fin_) can be used to quantitate the change of liposome topology and dynamics. Figure 5 shows values of (I_0,fin_−I_0,0_) parameter determined for various osmotic pressure differences and lipid bilayer compositions. Data shows that the outer interface does not depend on the value of the osmotic pressure difference. The value is practically invariant on the lipid bilayer composition, except the membrane containing lysoPC. In this case the change of scattered light intensity is the smallest. The (I_fin,fin_−I_0,fin_) values are different, they depend on both the lipid composition and on the osmotic pressure difference. Figure 6 shows (I_fin,fin_−I_0,fin_) values as a function of the osmotic pressure difference for membranes formed from various lipid mixtures.

When the lipid bilayer is formed from egg phosphatidylcholine alone, the change of the scattered light intensity does not depend on the osmotic pressure difference and its value is similar in magnitude to the value of (I_0,fin_−I_0,0_), showing that there is no significant membrane reorganization caused by the water outflow. When the lipid bilayer is formed from phosphatidylcholine mixed with 30 mol% cholesterol, values of (I_fin,fin_−I_0,fin_) are elevated by an order of magnitude indicating a massive alteration of the membrane molecular organization. The dependence of (I_fin,fin_−I_0,fin_) on the osmotic pressure difference shows that for all membranes, excluding those containing cholesterol, the extend of the scattered light intensity change increases with rising osmotic pressure difference, indicating increased changes in the membrane topology and properties. The value of (I_fin,fin_−I_0,fin_) determined for various membranes show that the introduction of compounds altering the lipid bilayer mechanics (cholesterol) or spontaneous curvature (lysoPC or phosphatidylethanolamine) result in increasing the parameter. The dependence of (I_fin,fin_−I_0,fin_) on the osmotic pressure difference may indicate that the final state of vesicles is a result of the balance between the extend of the membrane deformation, caused by the water outflow, and its mechanical resistance to that deformation inside vesicle. LysoPC and phosphatidylethanolamine should modify the pressure distribution along the membrane normal differently: lysoPC induces the positive spontaneous curvature whereas phosphatidylethanolamine favors the negative spontaneous curvature [59]. The presented experiments show that the two lipids affect the membrane in a similar way. The rising scattered light intensity (I_fin,fin_−I_0,fin_) can be interpreted as the alteration of the lipid bilayer topology and/or the different level of the water activity reduction.

### 2.1. The Probability of the Pore Formation and Membrane Ability to Fluctuate

**Figure 7:**
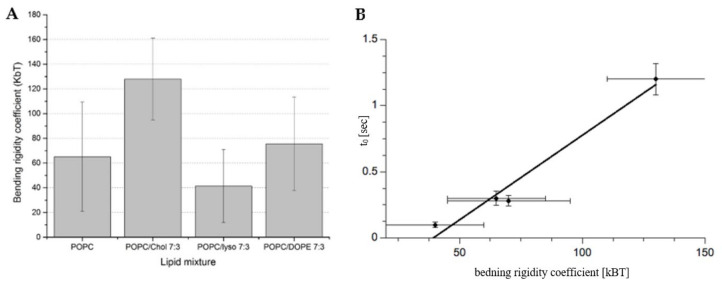
Plot (**A**) demonstrates the effect of different membrane compositions on their bending rigidity coefficient. ANOVA One-Way statistical test with 0.05 significance showed that populations PC-PC/chol 7:3 and PC-PC/lyso 7:3 are significantly different; there is no statistically significant difference when comparing PC with PC/PE 7:3. Plot (**B**) visualizes correlation between the probability of pore formation expressed by t_0_ and membrane bending rigidity coefficients.

The proposed soft-matter model of the lipid bilayer along with the experimental data supports the finding of the spontaneous transmembrane water pore formation across the lipid bilayer. This in turn should be related to the membrane mechanics according to Helfrich’s model (Equation (1)). Consequently, it can be predicted that the lipid bilayer propensity for topological transformation (lipid pore formation) correlates with the lipid bilayer mechanics, which can be quantitated using thermal fluctuations [60]. To verify the hypothesis, we used fluorescence enhanced flicker-noise microscopy method to measure the bending rigidity coefficients for the lipid mixtures studied. The method has been described in detail elsewhere [61]. The method is based on the fluorescence labeling of the GUV membrane, which can be used as an indicator of its homogeneity and improves the image analysis, as required for the flickering noise spectroscopy analysis. Bending rigidities of GUVs composed of POPC, POPC/chol 7:3, POPC/lysoPC 7:3 and POPC/DOPE 7:3 is presented in Figure 7. The obtained average values of bending rigidity expressed in k_B_T units for lyso, and cholesterol mixtures are in good agreement with previously published data. Measurement on mixture containing 30 mol% of phosphatidylethanolamine was performed for the first time and it did not differ significantly from phosphatidylcholine membrane. Figure 7B demonstrates the correlation between the membrane ability to fluctuate and the probability of the pore formation expressed by the t_0_ value determined using the stopped-flow experiment for a single osmotic pressure difference (Figure 3—1/ΔΠ = 0.05 mOsm-1). Values on the x-axis of the plot were calculated bending rigidity coefficient shown in Figure 7A.

The experimental data presented in the paper substantiate the model of the lipid bilayer, which accounts for the dynamics and organization of the lipid bilayer along with its dependence on properties of adjacent aqueous phases and can be conceptually represented in terms of soft matter physics. The understanding of molecular level processes, underlying the membrane permeability for water, will contribute to the understanding of the physiological role of lipid bilayer in maintaining intracellular homeostasis. Specifically, the effect of the cross-membrane osmotic imbalances on the membrane structural alteration demonstrates for the first time the correlation between the dynamics of the lipid bilayer and properties of adjacent aqueous phases [62]. It is believed that the biological space in general and the compositionally complex and heterogeneous cytoplasm in particular is a highly structured aqueous phase affecting properties of various supramolecular ensembles it contains [63,64]. It has been shown, for example, that the spatial distribution of ions at the membrane interface depends on type and quantities of charged lipids in the membrane as well as on their dipole moments [18,40,47,65,66]. It has been also shown that water structure and/or activity at a lipid surface differs from that in the bulk [47,67]. However, the effect of an aqueous phase on the lipid bilayer topology [47,68] has not been previously experimentally clearly demonstrated. There are data on the effect of water-soluble ions on the lipid bilayer state showing that there is mutual interdependence between membrane and the adjacent aqueous phase, and that perhaps even difference in water activities across lipid bilayer may cause the membrane topological alteration [62]. The aqueous phases separated by biological membranes in eukaryotic cells are usually osmotically balanced or the osmotic pressure differences are small and transient. When the osmotic pressure difference is significant, as demonstrated in the paper, the probability of the lipid pore opening increases, dissipating the trans-membrane proton gradient. It is difficult to envision how the concentration of the hydrophilic solute may induce the lipid pore formation but changing water activity at the interphase might. Consequently, it can be postulated that, where the water flow is a consequence of the osmotic imbalance across the membrane, the lipid pore opening is a consequence of the difference between water activities at respective interfaces. The water activity is also an important factor affecting the flow of protons across the biological membrane. The high permeability of the lipid bilayer for protons cannot be explained by the flux of large hydronium ions. However, the trans-membrane proton flow can be understood in terms of the Grotthuss mechanism, capable to transfer protons through water filled with lipid pores [69]. Therefore, the correlation between water and proton fluxes can serve as evidence of the existence of water filled pores in the lipid bilayer.

### 2.2. The Correlation between the Trans-Membrane Osmotic Pressure Difference and the Proton Flow

**Figure 8:**
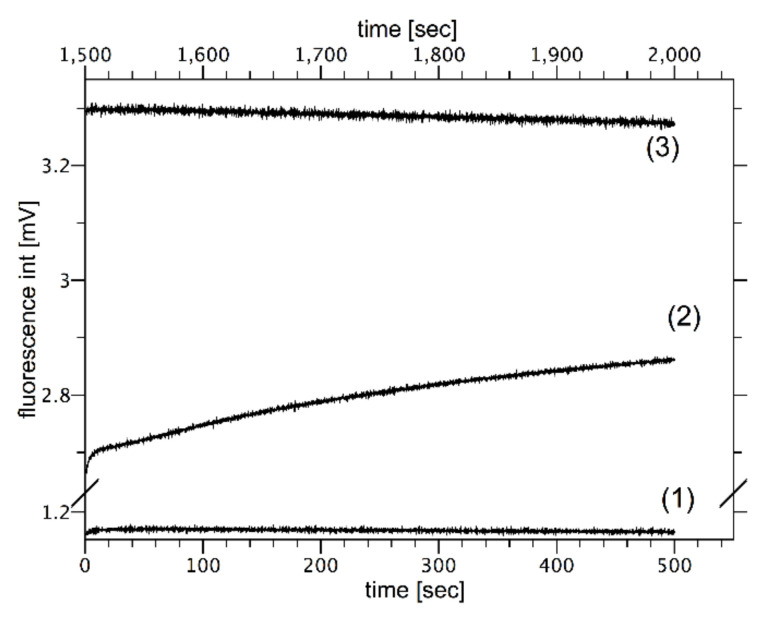
The kinetics of fluorescence intensity change for egg-PC vesicles labeled with fluorescein-PE exposed to the pH gradient ΔpH = 1.4 (kinetic 2) and at ΔpH = 0 (kinetic - 1) are presented. Plot 3 is plot 2 monitored in a different time interval (upper scale) showing the extant of the photo-bleaching effect. The pH gradient was generated by mixing vesicle suspension at pH 6.20 with buffer at pH 9 at proportions ensuring the desire pH gradient.

In order to measure the correlation between osmotic pressure difference and proton flow, liposomes labeled with fluorescein covalently attached to the lipid molecule were used. Due to the uniform labeling of the lipid bilayer, there are two distinct dye populations: one facing the outer and the other the inner aqueous phases. This makes it possible to monitor the proton concentration in water compartments separated by the lipid bilayer. When the liposome is simultaneously exposed to the osmotic pressure difference and pH gradients, the correlation between the rate of the volume change and the proton flux can be measured simultaneously. Figure 8 shows the time course of the fluorescence emitted by vesicles exposed to the pH gradient (ΔpH equals to 1.4) in isosmotic conditions (Curve 2). The fluorescence traces can be divided into two phases: the rapid fluorescence changes (within second) corresponding to the deprotonation of the fluorescent dyes located on the outer surface of the vesicle and extended in time fluorescence rises, which are correlated with the proton fluxes across the lipid bilayer. During the second phase, the fluorescence intensity changes only slightly, showing that the extend of pH gradients dissipation is insignificant during the experiment. The intensity monitored for the extended period of time when there are no pH gradients demonstrates that the photo-bleaching effect is irrelevant (fluorescence intensity changes by less than 3% in more than 3 h).

**Figure 9:**
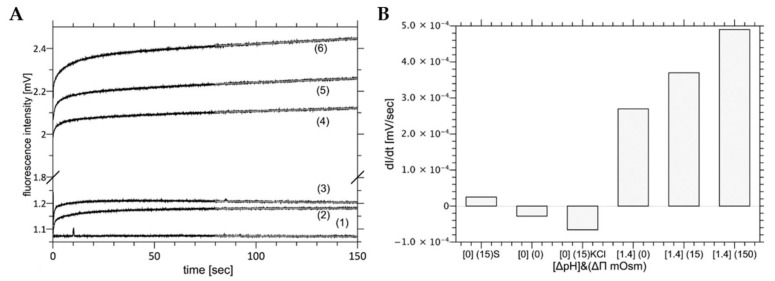
Panel (**A**) shows kinetics of fluorescence intensity changes caused by proton flow through liposome membrane exposed to different osmotic pressure differences at ΔpH = 1.4. Curve 4 represents the curve for Δπ = 0, Curve 5 for Δπ = 15 mOsm, and Curve 6 for Δπ = 150 mOsm. Curve 1 shows the fluorescence intensity when neither of gradients was present (ΔpH = 0 and Δπ = 0). Curves 2 and 3 represent the case when at ΔpH = 0 and Δπ = 15 mOsm. Curves 2 and 3 were generated with KCl or sucrose as osmotically active ingredients, respectively. Panel (**B**) shows slopes of the linear part of kinetics presented in panel A as a function of the osmotic pressure difference and/or pH.

Figure 9 shows selected kinetics of fluorescence intensity changes caused by proton flow through liposome membrane induced by ΔpH = 1.4 and when simultaneously exposed to osmotic pressure differences (Δπ = 0, Δπ = 15 mOsm, Δπ = 150 mOsm). The fluorescence intensity changes in time at ΔpH = 0 is also presented. Each curve consists of two kinetics: the fast process, which reflects the fluorescence change resulting from the protonation of dyes located at the outer interface of vesicles and the slow kinetics, which reflects the proton transfer across lipid bilayer. Since the proton flow across lipid bilayer is slow (hours), the size of proton flux (dI/dt) was approximated with linear function calculated from the fluorescence changes detected between 80–150 s following the exposure to pH and osmotic pressure gradients. Obtained values are presented in Figure 9B. In the case when no osmotic nor pH gradient were applied, the fluorescence intensity did not change in time (Curve 1). When vesicles were exposed only to the osmotic pressure difference (Δπ = 15 mOsm) the fluorescence intensity of fluorescein-PE increases slightly within 20 s following outer aqueous phase mixing, therefore, showing that the fluorescein-PE is weakly sensitive to properties of the membrane interface caused by water outflow, similar to results presented elsewhere [68,70]. There was no effect of the chemical nature of the osmotically active compound on the outcome of the experiment. However, when vesicles were exposed simultaneously to pH and osmotic pressure differences the fluorescence intensity increased in time, indicating pH changes inside the vesicles. The size of the fluorescence change is correlated with the value of the osmotic pressure difference (Figure 9B).

Data presented on Figure 9 can be interpret as follows: the osmotically induced water flow is facilitated by watered filled lipid pores. The lipid pore formation depends not only on intrinsic properties of the lipid bilayer, as described in the Helfrich model [71], but also on electrostatic properties of adjacent aqueous phases [30], and specifically on temperature or water activity. The membrane propensity for lipid pore formation increases with rising osmotic pressure difference, which also induces the elevated water flux requiring enhanced pores capacity as indicated by an increased proton flux. Since the nominal proton concentration gradient (<<1 mM) is negligible versus concentration of the osmotic active compound (150 mOsm), its effect on the water activity and, hence, probability of lipid pore opening is likely to be irrelevant. The observed slow fluorescence intensity changes shows that the intra-vesicle pH indicate the nominal cross-membrane proton flow and that the flow increases with rising osmotic pressure difference. This interpretation is consistent with the proposed molecular mechanism of water flow across the lipid bilayer so that the water channel connecting the two membranes’ separated aqueous phases is spontaneously formed.

## 3. Discussion

The scattered light intensity in the stopped-flow experiment has been used to monitor the evolution of liposome suspension exposed to osmotically pressure difference and flickering noise spectroscopy to evaluate the bending rigidity of the lipid bilayer. Stopped-flow experiments show that the kinetics of osmotically driven water flux out of liposome, at low osmotic pressure differences, consist of two phases separated by a deflection point. To quantitate the osmotically driven liposome shrinkage, the corresponding kinetics of scattered light intensity change was parametrized with the following parameters: the time of the deflection point (sec.) and the permeability coefficient derived from the slope of the curve following the deflection point. The character of the scattered light kinetics indicates that there are two processes involved in the water flux; spontaneous formation of the watered-filled lipid pores, which facilitate osmotically driven water flux. It can be hypothesized that each process is powered by different factors, dependent on the concentration of an osmotically active compound. Specifically, the water flow is driven by the difference in the concentration of osmotically active substances, whereas the lipid pore opening can be driven by the water activity gradient. The water flux, on the other hand, is an entropic effect driven by the osmotic pressure difference between membrane separate aqueous phases [72]. The spontaneous lipid pore formation can be energetically feasible, even without any external stress [30], since the energy required for its opening is of the order of a few tenths 10 k_B_T [30,72,73]. The probability of the lipid pore opening would depend on spontaneous curvature of the lipid bilayer and its components [30,31] and the Gaussian elastic modulus $\kappa_{G}$ according to the Helfrich representation of the membrane [26,74,75]. The membrane propensity for spontaneous lipid pore formation can be enhanced by system asymmetry, such as the difference in water activities in the adjacent aqueous phases [76]. The relevant water activity regions are dependent on the interface structure, which in turn depends on the composition of the lipid bilayer interface and the bulk water activity. This implicates that the composition of the aqueous phase will affect both the osmotic pressure and the water activity, each of the two parameters will influence different processes involved in the facilitation of the water flow across the lipid bilayer.

Experimental data presented in the paper leads to the new coherent perception of the lipid bilayer, which addressed a number of unresolved issues important for understanding molecular mechanisms of passive transport of hydrophilic substances across a lipid bilayer. The lipid bilayer can be treated as a soft dynamic matter, which intrinsic properties are responsible for its continuity, mechanics and permeability of hydrophilic compounds [75,77,78,79]. All these properties can be quantitated using the Helfrich model [26,27,28,29]. In addition, intrinsic properties of the lipid bilayer depend not only on its lipid composition but also properties of the adjacent aqueous phases (see for example [47,80]). This interdependence is of fundamental importance for the model of passive water transport across the lipid bilayer [46,81,82]. Based on the data presented in the paper and by others, the sequence of molecular events leading to water flow across the lipid bilayer can be proposed. When there is no concentration difference of osmotically active compound (membrane impermeable) across the lipid bilayer, the water exchange between compartments is slow [83]. In light of the proposed model, this results with the low probability of pore opening. The generation of the transmembrane concentration difference of two different gradients are formed: the osmotic pressure and water activity differences [54]. The water activity gradient will affect lipid bilayer interface, therefore, affecting the probability of the lipid pore formation [84]. This prediction is confirmed by the dependence of “t_0_“ on the osmotically active substance concentration gradient (Figure 3 and Figure 4). As expected, the “t_0_” is also dependent on temperature and the lipid bilayer composition. After pore opening the water flow is driven by osmotic pressure difference between aqueous phases and is only weakly dependent on the composition of the lipid bilayer (Figure 4). The osmotically driven water flow is halted by liposome resistance to deformation. The lipid pore enables the formation of a dynamic trans-membrane water bridge capable of facilitating, in addition to water flow, transfer of protons, which has far-reaching physiological consequences (Figure 9A) [57,85,86]. The probability of lipid membrane pore formation can be controlled by the membrane lipid composition [30,31]. With this respect, cholesterol seems to play a prominent role [87]. The dependence of the probability of pore opening on the osmotic pressure difference is negative feedback, allowing to dissipate any local osmotic pressure differences. When the mechanism is not sufficient, dedicated membrane proteins (aquaporins) are added to enhance membrane permeability to water [88]. Since lipid pores have relatively low selectivity, they dissipate transmembrane gradients, especially in regions where the concentration of osmotically active compounds frequently changes, as demonstrated by the experimentally demonstrated correlation between the osmotically induced water flow and the dissipation of the pH gradient. The dynamics of the lipid bilayer exposed to the difference in concentration of osmotically active compound can be adequately described by the Helfrich model [26], where the membrane intrinsic properties such as bending rigidity coefficient, spontaneous curvature or gaussian elastic modulus are sufficient to describe the effect of membrane properties on the water and proton flux across the lipid bilayer. The specific arrangement of lipid molecules within and near the pore opening cannot be elucidated from the experimental data presented in the paper alone. However, based on computer simulations, it can be hypothesized that pore is formed by hydrated polar lipid headgroups. Such arrangement will facilitate the mobility of water molecules required for proton transfer but prevent the flow of hydrated solutes [41,69,89].

Furthermore, the water flow can be represented as the two-stage process: the water activity gradient dependent lipid pore opening and osmotically active compound concentration difference drove water flow.

## 4. Materials and Methods

### 4.1. Chemicals and Liposome Preparation

Phosphatidylcholine (PC), lyso-phosphatidylcholine (lysoPC), cholesterol (chol) and phosphatidylethanolamine (PE) were purchased from Avanti Polar Lipids (Amsterdam, Netherlands) and were of analytical grade. Fluorescent probe, fluorescein-PE and carboxy-fluorescein (>99%) were purchased from Molecular Probes (Eugene, OR, USA). All experiments were carried out in the deionized water (conductivity less than 0.1 µS). In order to generate the osmotic pressure difference across the lipid bilayer the water solutions of KCl, NaCl or sucrose (POCH, Gliwice, Poland, 99.99%) were used. Liposomes were prepared by the extrusion method [90]. In short, the appropriate amounts of lipids dissolved in chloroform were mixed together (when needed, with the addition of 0.1 mol% of fluorescein-PE) and the organic solvent was than evaporated using the stream of nitrogen. To remove the remnants of the organic solvent, the lipid film was left overnight under vacuum. The resulting dry lipid film was hydrated and agitated for 2 h to obtain milky multilamellar vesicles suspension. Next, the suspension was extruded through the 100 nm polycarbonate membrane filters (Whatman, Maidsone, UK). In samples where the entrapment of carboxy-fluorescein was needed, the dry lipid film was hydrated with aqueous phase with the fluorescent dye (0.05 M) added. The non-encapsulated dye was removed by passing the vesicle suspension through the column (Sephadex 25G; Cytiva, Marlborough, MA, USA), as described in detail elsewhere [45]. The quality of the liposome suspension was tested prior and after each measurement using the dynamic light scattering technique (NanoSizer ZS, Malvern, UK). Typically, the average vesicle size was equal to 110 nm ± 10 nm with the polydispersity index always smaller than 0.12. Liposome size depended on the lipid type and composition to the small extent. Vesicles size distribution was also measured as a function of pressure difference and time after application of the osmotic pressure difference. The pH of buffer solutions for proton transport measurements were controlled with pH-meter, calibrated for pH 4, 7 and 11 prior each measurement.

### 4.2. Determination of the Water Flux across the Lipid Bilayer

The kinetics of the osmotically induced water flow was measured with the stopped-flow technique using the scattered light as an indicator of vesicles shrinkage. The wavelength of the incident beam was set to 405 nm. The stopped-flow instrument (BioLogic, Grenoble, France) was equipped with the xenon-mercury lamp, the monochromator with 0.5 nm slit and the quartz flow cuvette with 1.5 mm optical path. The liposome suspension in buffer (~10 mOsm) was mixed with osmotically active (membrane impermeable) solutions with different osmolality (between 30 mOsm and 300 mOsm) in 1 to 1 volume ratio. This generates the osmotic pressure difference across the liposome membrane (in the range between 15 mOsm and 150 mOsm). We have demonstrated previously that the intensity of light scattered by liposomes is affected predominantly by the state of lipid bilayer interfaces. When exposed to the osmotic pressure difference, membrane interfaces are affected by rising concentration of entrapped compounds and/or by altered vesicle shape [91]. In order to provide the evidence for the qualitative correlation between the intensity of scattered light and the change of the liposome volume, the experiment using entrapped fluorescent self-quenching dye was performed. In this case, the vesicle shrinkage, induced by the osmotic pressure difference, was monitored using both the scattered light and the fluorescence emission of the encapsulated carboxy-fluorescein at the same experimental conditions. Experimental values assigned to relevant time points form a straight line showing that there is a direct correlation between scattered light intensity and the size of vesicle internal volume, as judged by the extend of the self-quenching of encapsulated carboxy-fluorescein [91]. Nevertheless, rising the scattered light intensity will more likely result from the refractive index alteration due to the interface reorganization of the lipid bilayer, upon shrinkage rather than the change of lipid aggregate size. Small decrease in hydrodynamic radium upon water outflow (10% of initial size), as measured by DLS along with predictions from the Lorenz–Mie theory, supports this finding, as it was already discussed elsewhere [91].

### 4.3. The Kinetics of the Transmembrane Proton Flux

**Figure 10:**
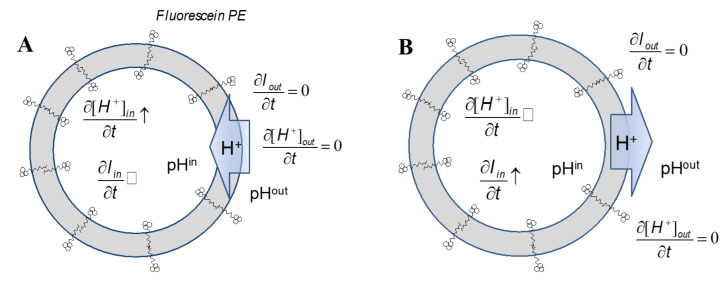
The drawing demonstrates an experimental design for proton transfer measurements using fluorescein-PE. In general, higher proton inner concentration (lower pH—panel **B**) will result in proton efflux and the decrease of the fluorescein intensity, while the lower proton inner concentration (higher pH—panel **A**) outcomes with the fluorescence increase.

Assuming that proton transfer in aqueous phase is facilitated mainly by Grotthuss mechanism, which requires that the proton transfer requires a continuous chain of mobile water molecules [69]. Consequently, it has been assumed that any observed proton transfer across the lipid bilayer should implicate the existence of water-filled lipid pores [29,92]. The proton transfer across lipid bilayer was monitored with stopped flow method, described in detail elsewhere [93]. In short, fluorescence intensity changes of pH sensitive fluorescein-PE were measured as a function of time for a given pH gradient (Figure 10). The pH sensitive fluorescein moieties, covalently attached to the lipid head-group, consist of two dye populations located on the opposite sides of the lipid bilayer and do not contribute to the intra-vesicle osmotic pressure. The two populations of dyes sense the pH values in aqueous compartments separated by the lipid bilayer. To confirm the existence of lipid pores, the proton and water fluxes across the lipid bilayer were induced by exposing vesicles to both the osmotic pressure and pH gradients. Specifically, liposomes extruded in buffer at pH 9.0 were mixed with buffered potassium chloride solution at pH 5.0. Such combination of aqueous phases results in two fluxes: water flux out of vesicles, induced by the osmotic pressure difference and the proton transfer driven by the pH gradient. The kinetics of water flow was evaluated by the extend of scattered light intensity change, whereas the proton flux with the change of the fluorescence intensity of membrane-associated fluorescent dyes [93]. When vesicle suspension is exposed to the pH gradient at the presence of osmotic pressure difference, the change of the fluorescence intensity is affected by local pH and optical properties of the vesicle suspension. The latter factor was evaluated when vesicles were exposed to 15 mOsm gradient in the absence of pH gradient. In this case the resulting fluorescence change has not exceeded 5% of the value when pH gradient was present. This shows that the osmotic pressure difference does not affect the intensity of fluorescence emitted by fluorescein-PE dyes. Proton flux across the lipid bilayer can be facilitated by two different mechanisms: a simple diffusion of hydronium ions or the charge transfer by a co-operative motion of water molecules (Grotthuss effect) [94]. The diffusion of a hydronium ion across the lipid bilayer is unlikely since its size and the net electrostatic charge is similar to that of the hydrated sodium ion, which is not capable to cross the hydrophobic barrier of the lipid bilayer [29]. The Grotthuss mechanism is a very efficient process because it relies on the coordinated motion of water molecules (water mobility correlates with the lifetime of the hydrogen bonds, which the surfaces of hydrated lipids are of the order from 10^−10^ s to 10^−12^ s) [94]. However, in order to facilitate the proton transfer across the lipid bilayer, the Grotthuss mechanism, which requires a continuous and dynamic water bridge connecting aqueous phases, has been implicated. Consequently, there should be a correlation between water flux and proton transfer across the lipid bilayer, which can be, at least qualitatively, experimentally tested.

### 4.4. Bending Rigidity Coefficient Determination Using Flicker-Noise Spectroscopy for POPC, POPC/chol 7:3, POPC/lysoPC 7:3 and POPC/DOPE 7:3 Mixtures

An electroformation method was used for model lipid membranes formation. 15 µL of 1 mg/mL mixtures were deposited in small quantities onto platinum electrodes. Electrodes were kept under low pressure for 1 h to remove organic solvent (chloroform). This was followed by immersing electrodes in aqueous solution and exposing them to 1 Hz AC electrical field for 4 h with increasing voltage from 1 V to 4 V. Next, electroformation chambers were left for 1 h to allow descent of vesicles from electrodes.

Images of vesicles were collected using Leica TCS SPE microscope in fluorescence mode equipped with 63x/1.30 ACS APO oil-immersion objective (Leica, Wetzlar, Germany). 1392 × 1040 pixels images were recorded with Leica DFC310 FX camera using 0.102 μm pixel size. Samples were illuminated with fluorescence illuminator EL6000 and fluorescence emission was detected using N2.1 cut off filter. GUVs prepared for microscope were labelled with 1 m% Rhodamine-PE. Series of images were collected for 5 min with 33.4 fps resulting in total 10,000 frames, which were then subjected to image analysis and contour extraction (after contour extraction on average 7500 frames were used for bending rigidity coefficient determination).

## 5. Conclusions

The experimental data presented in the paper have been used for the formulation of the conceptual model of the water and proton fluxes across a lipid bilayer. In the model, the dynamics of lipid molecules play a critical role not only in the transport of water across the lipid bilayer but also in overall membrane mechanics. Spontaneously formed lipid pores facilitate osmotically driven water flow through the unmodified lipid bilayer. The dependence of the propensity of the lipid pore formation on the lipid composition justifies the assumption that the parameter can be considered as an intrinsic property of a lipid bilayer. When the osmotic pressure difference across the membrane is small (tens of mOsms), the water flux does not follow the simplistic model, as water flows through the uniform semipermeable membrane. Specifically, there is a time period immediately following the osmotic pressure exposure when no vesicle size change was observed. This delay might be correlated with the time needed for the membrane lipids reorganization necessary for the lipid pore formation. In another experiment, it has been demonstrated that the presence of water-filled lipid pores facilitates the proton flow across the hydrophobic core of the membrane indicating the physiological significance of the observed phenomena. In addition, the correlation between the vesicle resistance to deformation caused by osmotic imbalance and bending rigidity coefficient shows that the two properties have the same origin namely, dynamics of lipid molecules in the lipid bilayer.

## Data Availability

The data presented in this study are available in the article.
